# DigDig: A Software for In-Depth Analysis and Comparison of Proteolytic Digestion

**DOI:** 10.1021/acs.analchem.5c04217

**Published:** 2025-09-25

**Authors:** Zuzana Kalaninová, Jasmína Mária Portašiková, Daniel Kavan, Petr Novák, Petr Man

**Affiliations:** † Department of Biochemistry, Faculty of Science, 112302 Charles University, Hlavova 8, Prague 2, Prague 12843, Czech Republic; ‡ Institute of Microbiology of the Czech Academy of Sciences, BioCeV, Videnska 1083, Prague 4, Prague 14220, Czech Republic

## Abstract

Proteolysis is a crucial step in both bottom-up and structural proteomics workflows, directly influencing peptide identification and sequence coverage in mass spectrometry-based analyses. While classical proteomics typically relies on highly specific enzymes with well-defined cleavage patterns, structural MS approaches such as hydrogen/deuterium exchange mass spectrometry (HDX-MS) often employ nonspecific or semispecific proteases, producing complex peptide mixtures that require more detailed digestion analysis. To address these needs and streamline the entire process, we developed DigDig, a standalone, Java-based software tool for evaluating and comparing proteolytic digestion across diverse experimental conditions. DigDig processes output files from common search engines and provides customizable visualizations of key digestion metrics, including sequence coverage, reproducibility, peptide redundancy, cleavage site preferences, and peptide length distributions. A distinguishing feature is its ability to detect and report repetitive peptide sequences, which are frequently missed by standard tools. We demonstrate its capabilities using data sets from both specific and nonspecific digestions, highlighting its utility in digestion quality control, protease characterization, and method development, particularly in HDX-MS workflows. DigDig is freely available at https://peterslab.org/DigDig/.

## Graphical abstract

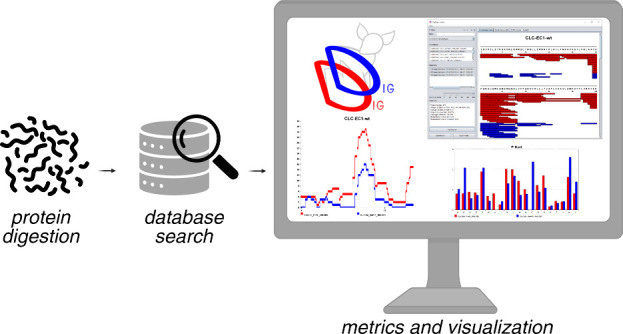

## Introduction

Proteolysis is an essential step in bottom-up proteomics workflows, where proteins are enzymatically cleaved into peptides prior to mass spectrometric analysis.^1–3^ The efficiency and reproducibility of digestion significantly impact the accuracy of peptide identification, quantification, and overall data interpretation. In classical proteomics, highly specific enzymes like trypsin are typically employed due to their well-defined cleavage pattern.^4^ In contrast, structural proteomics workflows, particularly hydrogen/deuterium exchange mass spectrometry (HDX-MS), rely on nonspecific or semispecific proteases,^5–10^ which exhibit broader specificities and produce many overlapping peptides. While this enhances the spatial resolution of the experiment, it also introduces challenges in digestion predictability and downstream data processing. Search engines such as MASCOT,^11^ PEAKS,^12^ FragPipe^13^ or MaxQuant^14^ typically report digestion-related metrics including sequence coverage, peptide counts, length distribution, and the number of missed cleavage sites (applicable to specific digestions). In addition, postprocessing tools like IceLogo^15,16^ offer probability-based analysis and visualization of cleavage site preferences in a sequence logo. SPACEPro^17^ provides insight into digestion efficiency by analyzing mis- and nonspecific cleavages at the peptide-spectrum match (PSM), peptide, and protein levels in shotgun proteomics MS data.

However, in HDX-MS, particularly during its feasibility stage, it is essential to compare a wide range of digestion conditions, including different proteolytic columns, denaturing and reducing conditions, flow rates, and temperatures. Likewise, the development of novel proteases calls for deeper insight into digestion characteristics and cleavage preferences. To address these needs, we developed DigDig, a standalone, Java-based software tool. Based on search engine outputs, DigDig calculates a set of digestion metrics, including sequence coverage, redundancy and peptide length distribution, and cleavage site profiles, and presents them through multiple, user-friendly, customizable visualizations. We demonstrate its utility using data sets from both specific and nonspecific digests, highlighting how DigDig supports routine digestion quality control, method optimization, and protease characterization not only in HDX-MS but across diverse bottom-up proteomics workflows.

## Experimental Section

DigDig is distributed as a standalone Java archive (*.jar) file and does not require installation. To run the software, any recent LTS version of a standard Java Virtual Machine (JVM) must be installed (e.g., Oracle JDK 21 as of August 2025). Up-to-date instructions are provided on the DigDig wiki page, section ***Installing and running*** - https://peterslab.org/DigDig/doc/doku.php?id=standardrun). In addition, the system path to correct java.exe must be included in the system’s environment variables (PATH), which is also explained in detail in the wiki documentation. This wiki-based documentation, available at https://peterslab.org/DigDig/, includes installation instructions, descriptions of all functional modules, and example data sets used in this study, as well as additional test data for tutorial purposes. The data presented here were generated de novo specifically to ensure comprehensive coverage of all aspects outlined in the manuscript. A detailed description of the methods is provided in the Supporting Information.

The software is fully compatible with search results exported in *.csv format from MASCOT and PEAKS. Output files from Byonic, Waters PLGS, and SpectroMine are also supported. The additional input file required for full functionality is the corresponding FASTA database used during the search.

## Results and Discussion

Upon executing the DigDig jar file, a dialogue window appears prompting the user to select the corresponding database (FASTA file) used in the original search. In the next step, the user selects the search result files for processing. If the recommended naming convention is followed, DigDig automatically recognizes replicates and experimental conditions. This recognition is based on the position of the last underscore in the filename: files sharing identical text before the last underscore are grouped as replicates. Currently, this is the only method available for sorting data within the program, though additional options are planned for future versions. After loading the files, DigDig matches the search results to entries in the database and detects repetitive sequences. These repetitions are displayed in a separate window and can be exported as a standalone file. This functionality is described in more detail below. Data analysis in DigDig proceeds via two main pathways - processing at the level of individual proteins (*Coverage Map* and *Redundancy Plot*) or at the level of all or selected protein(s) (Cleavage Preferences and Peptide Length Distribution). This dual structure is reflected in the organization of the left sidebar, which serves as the main navigation element across both analysis modes. It should be explicitly noted that the current version of DigDig does not account for peptide modifications. Modified peptides are not excluded from the visualizations but are treated in the same way as their unmodified counterparts. Support for peptide modifications is among the planned features, which will enable users to assess the effect of specific modifications on a cleavage pattern.

### Sidebar for Coverage Map and Redundancy Plot

The top window displays all identified proteins, which can be sorted either alphabetically (A–Z) or by the number of identified peptides in descending order. A *Filter* field allows users to search for specific proteins by name, as defined in the FASTA file headers. The *Conditions* field lists experimental conditions, which are automatically grouped based on file naming conventions. The *Analyses* field shows individual analyses associated with the selected condition, along with the number of identified peptides in each of them. Both fields support multiselection, enabling the pooling of several conditions and/or analyses. To assess data consistency, users can adjust the *Reproducibility* level, which ranges from 0% (all peptides from all analyses are included) to 100% (only peptides found in all replicates are used for processing). The final panel, *Statistics*, displays digestion metrics calculated from the selected data and adjusted according to the chosen reproducibility level. Several parameters are reported. *Protein length* is derived from the corresponding database (FASTA) entry. The *Unique peptide number* indicates the number of peptides that pass the filtering criteria out of all unique peptides identified across the selected conditions and analyses. This value, along with the corresponding percentage, provides a basic measure of digestion reproducibility. *Average peptide length* offers a rough estimate of peptide size, while a more detailed view is available under the *Length* tab, which shows the full peptide length distribution. *Sequence coverage* reflects the proportion of the protein sequence covered by the identified peptides. *Cleavage efficiency* is calculated based on the number of cleaved peptide bonds relative to the theoretical total. And finally, the *Redundancy score* quantifies how often each residue is covered, either across the entire protein sequence or restricted to peptide-covered regions. This value represents an average, a more informative, position-specific view is available under the *Redundancy Plot* tab.

### Sidebar for Preferences and Lengths

Similar to the previous section, the left sidebar is used to select data for visualization and data export. The *Protein* field now allows selection of one or multiple entries. The *Conditions* and *Analyses* fields function as described earlier. Unlike the sidebar used for the *Coverage Map* and *Redundancy Plot*, all fields here support single, multiple, or full selection. This enables pooling across several or all proteins, conditions, and analyses. As before, the *Reproducibility level* can be adjusted to filter peptides based on their occurrence across replicates.

### Data Visualizations

Once the data are prepared, DigDig provides several visualization options available under separate tabs: Coverage Map, Redundancy Plot, Cleavage Preferences, and Peptide Length Distribution (Figure 1). Each visualization can be exported in *.pdf, *.png, or *.svg format. Additionally, the underlying data can be saved as a plain text file for further analysis or custom processing.

**Figure 1:**
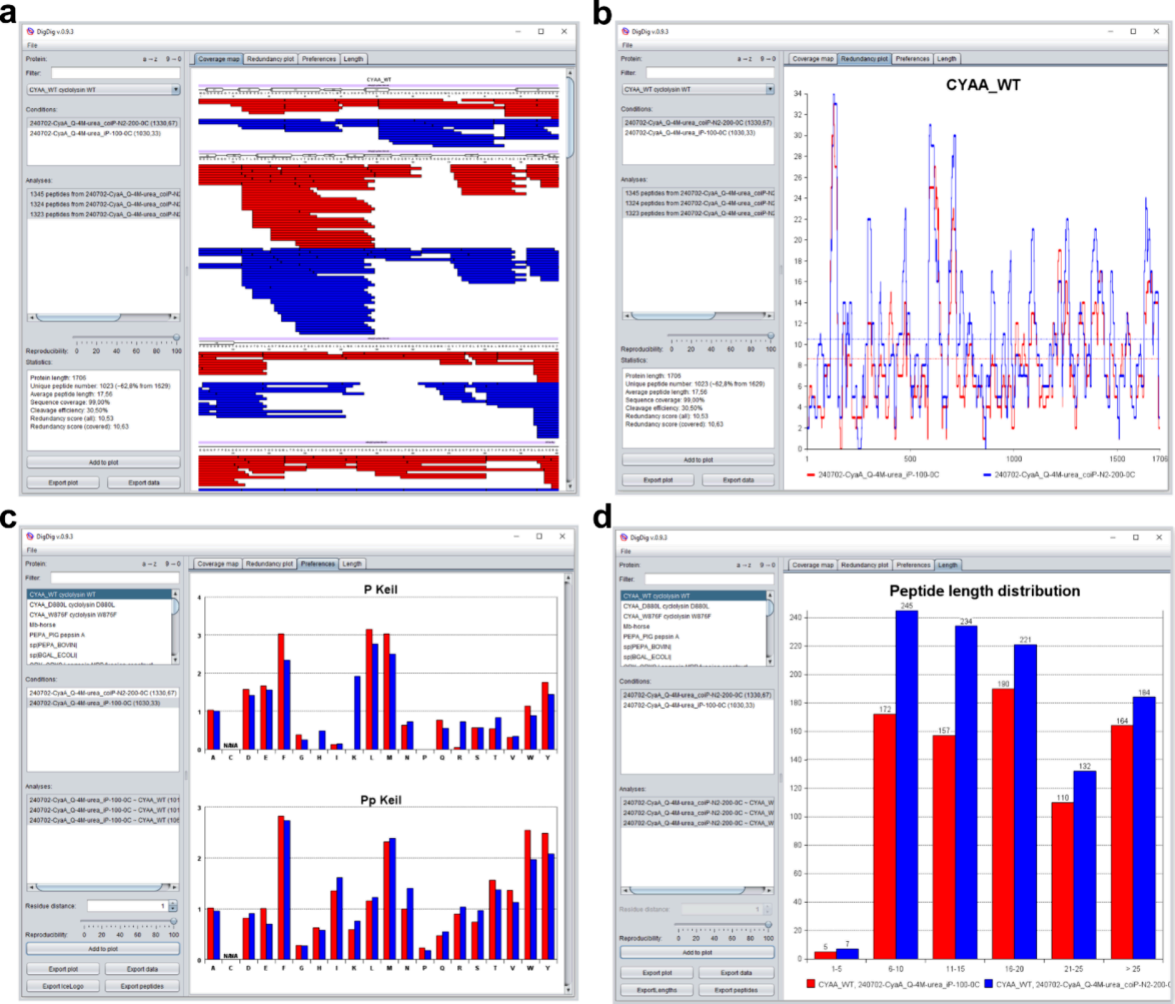
Visualization options in DigDig used to compare the digestion of *Bordetella pertussis* adenylate cyclase toxin (CyaA) with immobilized pepsin (red) and coimmobilized pepsin and nepenthesin-2 (blue). All plots were generated using fully reproducible peptides (identified in 3 out of 3 analyses). (a) Coverage Map and (b) Redundancy Plot illustrate sequence coverage and residue-level redundancy across the entire protein. (c) Cleavage Preferences reveal the protease-specific cleavage pattern and highlight potential differences under varying digestion conditions. (d) The peptide length distribution provides an overview of peptide size and contributes to the overall assessment of digestion.

The *Coverage Map* (Figure 1a) visualizes protein sequence coverage following digestion, enabling comparison across selected experimental conditions. The layout is fully customizable via the settings window, which can be accessed by right-clicking within the map area. Users can adjust parameters such as the number of amino acids per line, band height, border and color settings, font style, residue numbering, and numbering interval. Additionally, secondary structure elements and functional domains can be overlaid on top of the sequence for enhanced interpretability.

Another visualization option is the *Redundancy Plot* (Figure 1b), which presents similar information as the Coverage Map but compressed into a single line, making it more suitable for comparing multiple conditions or for large proteins (such as CyaA, ∼1700 amino acids). The *x*-axis represents the protein sequence, while the *y*-axis indicates redundancy, that is, how many times the individual peptides cover each amino acid. In an ideal tryptic digest with nonoverlapping peptides covering the entire sequence, the plot would form a flat line at a value of 1. In contrast, nonspecific digests with overlapping peptides produce a fluctuating profile. As with the Coverage Map, the plot’s appearance can be customized, including line color and style.

The *Cleavage Preferences* analysis enables comparison of protease cleavage patterns or the effects of different digestion conditions. The cleavage preferences are calculated based on the identified peptides and the data are normalized according to Keil:^18^

$${\mathrm{CS}}_{\mathrm{norm}}=\left(\sum \left({\mathrm{Px}}_{\mathrm{AA}}\right)/\sum \left({\mathrm{Px}}_{\mathrm{tot}}\right)\right)/\left(\sum \left({\mathrm{seq}}_{\mathrm{AA}}\right)/\sum \left({\mathrm{seq}}_{\mathrm{tot}}\right)\right)$$

where CS_norm_ stands for a normalized cleavage specificity; Px_AA_ is the number of occurrences of an individual amino acid in Px position with x limited to a maximum of 10; Px_tot_ is the total number of identified cleavage sites; seq_AA_ is the number of occurrences of an individual amino acid in all protein substrate sequences; and seq_tot_ is the total number of residues in all protein substrate sequences. The DigDig calculates the preferences up to the tenth position (P10) from the cleavage site. The preferences can be extracted from one, multiple selected, or all identified proteins. Here we demonstrate the extraction of cleavage preferences on an example of CyaA that was digested online by immobilized pepsin or using coimmobilized pepsin and nepenthesin-2 (Figure 1c). As expected, pepsin preferentially cleaves after Phe, Leu, and Met at the P1 position,^5^ while cleavage after Pro and basic residues (His, Lys, and Arg) is disfavored. In contrast, the broader substrate specificity of nepenthesin-2 ^10^ results in a wider distribution of cleavage sites. This analysis is particularly informative for nonspecific or semispecific proteases, but it can also reveal unexpected cleavage preferences for specific enzymes when using ″no enzyme″ search settings. One notable example is the detection of a cleavage preference for unmodified cysteine by prolyl endoproteases, which is not observed when cysteines are alkylated, oxidized, or involved in disulfide bonds.^19^ Another example is the tryptic digestion of either a single protein (bovine serum albumin, BSA) or a complex mixture (HEK293 cell lysate), analyzed using *no enzyme* search mode. Cleavage preferences were extracted for BSA alone or for all identified proteins in the HEK293 lysate (Figure 2). Although trypsin is described as highly specific for Arg and Lys,^4^ detailed analysis of the single protein digest revealed a notable number of semispecific peptides^20^ leading to a rather broad cleavage specificity pattern (Figure 2a). In BSA digestion, 96 fully tryptic peptides (73 with at most one missed cleavage) and 145 semispecific peptides were identified, and no nonspecific peptides were observed. While the semispecific peptides accounted for ∼60% of identifications, most were of a low abundance (Figure S1). On the other hand, with highly complex protein samples such as HEK293 cell lysate, the specificity is higher (Figure 2b), which aligns well with the previous studies.^4,20^

**Figure 2:**
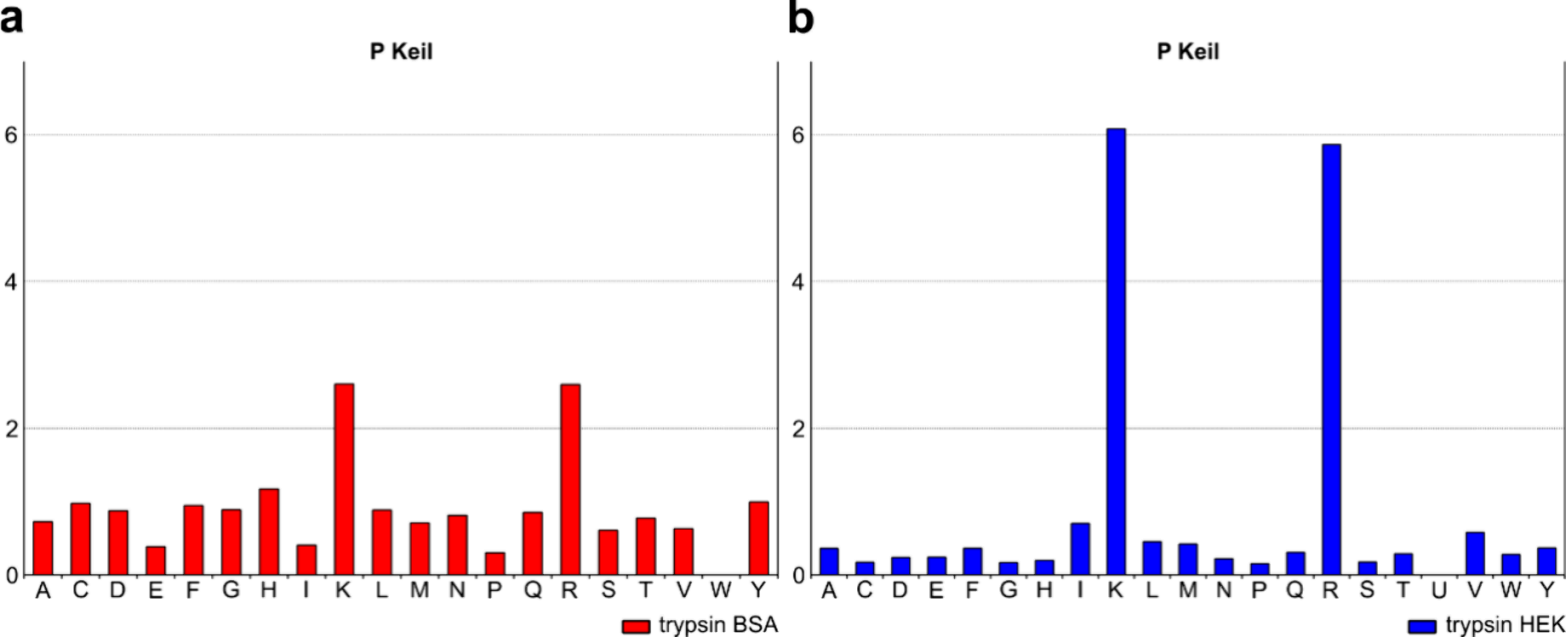
Extraction of cleavage preferences from test mixtures. Cleavage preferences at P1 position were calculated from the no-enzyme search results of (a) BSA and (b) HEK293 cell lysate. Only fully reproducible peptides (3 of 3) were used.

An additional feature of the *Cleavage Preferences* tab is the ability to export data, specifically the list of sequence stretches, that can be readily uploaded into the IceLogo web server (https://iomics.ugent.be/icelogoserver/create).^15,16^ The final visualization, *Length*, extends the basic “average peptide length” metric by displaying the full distribution of peptide lengths within the data set (Figure 1d). Similar to *Cleavage Preferences*, peptide length distributions can be extracted for a single protein, a selected group, or all identified proteins. Binning is symmetric by default and can be customized via the settings window.

An additional feature of DigDig, also mentioned above, is its ability to detect peptide sequences that occur repetitively within the target protein. While this is relatively rare with specific digestion, nonspecific proteases often produce shorter peptides whose sequences may not be unique. We encountered this issue while analyzing human haptoglobin, namely allele 2, which contains nearly identical sequence repeats resulting from gene duplication.^21,22^ We also observed that most search engines handle such repetitive sequences inconsistently and often overlook additional occurrences of nonunique peptides (Figure 3). To investigate this further, we searched a myoglobin digest against a database containing four concatenated copies of the myoglobin sequence (Figure S2). Based on the results, search engines either fail to recognize or assign peptides to repeated regions (Figure S3–S9), resulting in lower apparent sequence coverage (MASCOT,^11^ PEAKS,^12,23^ Byos)^24^ or they acknowledge the repetition (e.g., in visualizations) but do not list repeated peptides explicitly in the output (ProLuCID,^25^ FragPipe,^13^ MaxQuant,^14^ novor.cloud)^26^ (Figure S3). Here, DigDig provides a solution by identifying all occurrences of repeated peptides, providing the user with their detailed descriptions, and displaying them in the sequence coverage map. Because repetitive sequences cannot be unambiguously assigned to a single position, their interpretation remains debatable. To address this, we implemented an option that allows users to directly compare results with repetitive sequences either included or completely excluded. This functionality will be further developed in the future versions of the software.

**Figure 3:**
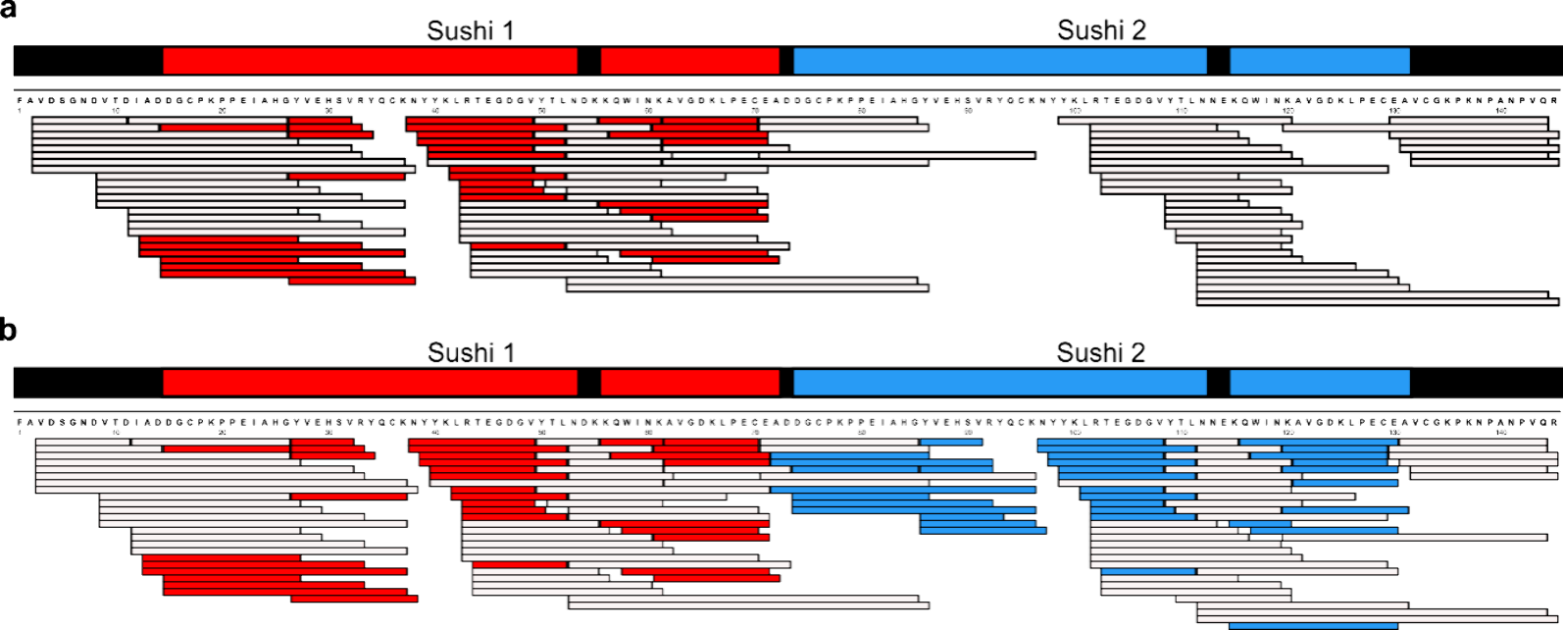
The occurrence of repetitive peptide sequences is overlooked by the search engines. Amino acid repeats in α-chain of human haptoglobin 2 visualized using MASCOT (a) and DigDig (b). Repetitive peptides colored in red (first occurrence) and blue (second occurrence).

## Conclusions

We presented DigDig, a new software tool designed for fast yet comprehensive analysis of proteolytic digestion. DigDig enables in-depth evaluation of digestion reproducibility, cleavage specificity, and peptide coverage across diverse experimental conditions. It provides visualization tools optimized for both specific and nonspecific digestion workflows, addressing a key limitation of conventional search engines by detecting and reporting repetitive peptide sequences. With its user-friendly interface, customizable outputs, and broad compatibility with search engine results, DigDig supports a wide range of applications, including HDX-MS method optimization, protease characterization, and routine quality control in bottom-up proteomics.

## Supplementary Material



## Data Availability

All data are available for download at https://peterslab.org/DigDig/. Source code can be obtained from the authors upon written request.

## Notes

Additional methods (online digestion of CyaA and LC-MS/MS analysis, trypsin digestion and LC-MS/MS analysis of BSA and HEK293 cell lysate, and online digestion of myoglobin and haptoglobin 2-2 and LC-MS/MS analysis); extracted ion chromatograms of tryptic peptides identified from a bovine serum albumin digest; sequence coverage of concatenated myoglobin; handling of repetitive peptide sequences by various search engines; and search results of sequence coverage from MASCOT - BioPharma Compass, PEAKS Studio, Byos, ProLuCID - ProteoScape, FragPipe, and novor.cloud (PDF)
