# Supplementary material for: DigDig: A Software for In-Depth Analysis and Comparison of Proteolytic Digestion
Source: Anal Chem. 2025 Sep 25;97(39):21205–10. doi: 10.1021/acs.analchem.5c04217 (PMC12509190; doi:10.1021/acs.analchem.5c04217)
Supplement: Supplementary file 1 [file ac5c04217_si_001.pdf]

Supporting Information

DigDig: A Software for In-Depth Analysis and Comparison of Proteolytic Digestion

Zuzana Kalaninová[†,‡], Jasmína Mária Portašiková[†,‡], Daniel Kavan[‡], Petr Novák[†,‡], and Petr Man[*,‡]

[†] Department of Biochemistry, Faculty of Science, Charles University, Hlavova 8, Prague 2, 12843, CZ

[‡] Institute of Microbiology of the Czech Academy of Sciences, BioCeV, Videnska 1083, Prague 4, 14220, CZ

*Corresponding author - pman@biomed.cas.cz

The content in supporting information

Methods:

On-line digestion of CyaA and LC-MS/MS analysis

Trypsin digestion and LC-MS/MS analysis of BSA and HEK293 cell lysate

On-line digestion of myoglobin and haptoglobin 2-2 and LC-MS/MS analysis

Figures:

Figure S1 – Extracted ion chromatograms of tryptic peptides identified from a bovine serum albumin digest.

Figure S2 – Sequence coverage of concatenated myoglobin.

Figure S3 – Handling of repetitive peptide sequences by various search engines.

Figure S4 – Search result – sequence coverage from MASCOT – BioPharma Compass

Figure S5 – Search result – sequence coverage from PEAKS Studio.

Figure S6 – Search result – sequence coverage from Byos.

Figure S7 – Search result – sequence coverage from ProLuCID - ProteoScape.

Figure S8 – Search result – sequence coverage from FragPipe.

Figure S9 – Search result – sequence coverage from novor.cloud.

Supporting methods

On-line digestion of CyaA and LC-MS/MS analysis

RTX toxin CyaA was prepared as described previously[1,2]. Protein in 8M urea, 50mM Tris-HCl pH 8.0 was diluted with 50 mM HEPES (pH 7.4), 150 mM NaCl, and 4 mM CaCl2 buffer to initiate folding. The following steps were conducted by the PAL DHR robot (CTC Analytics AG, Zwingen, Switzerland) controlled by Chronos software (AxelSemrau, Sprockhoevel, Germany). To mimic the HDX-MS experiment, protein at the concentration of 5 µM was further diluted with 50 mM HEPES (pH 7.4), 150 mM NaCl, 2.4 M urea, and 4 mM CaCl2. Next, sample was mixed with 1 M glycine-HCl (pH 2.3), urea (5.6 M) quench buffer at 1:1 (v/v) ratio and immediately injected into the LC system. The system consisted of a temperature-controlled box and Agilent Infinity II UPLC (Agilent Technologies) coupled to an ESI source of timsTOF Pro with PASEF (Bruker Daltonics). The LC setup was cooled to 0 °C and consisted of immobilized pepsin or co-immobilized pepsin-nepenthesin-2 custom-made proteolytic column (bed volume 70 µl), trap column (SecurityGuard ULTRA Cartridge UHPLC Fully Porous Polar C18, 2.1 mm ID, Phenomenex), and an analytical column (Luna Omega Polar C18, 1.6 µm, 100 Å, 1.0 × 100 mm, Phenomenex). Samples were digested and peptides desalted by 0.4% formic acid (FA) in water delivered by the 1260 Infinity II Quaternary pump either at 100 µl/min (for pepsin) or 200 µL/min (for mixed column). Water-acetonitrile gradient (10%–45%; solvent A: 0.1% FA in water, solvent B: 0.1% FA, 2% water in acetonitrile) followed by a step to 99% B was used to elute and separate the desalted peptides. The solvents were driven by the 1290 Infinity II LC system pumping at 40 µl/min. Mass spectrometer operated in data-dependent MS/MS mode with the tims activated. The LC-MS/MS data were peak picked in DataAnalysis (Bruker Daltonics), exported to text files, and searched using MASCOT (v 2.7, Matrix Science) against a custom-built database combining a common cRAP.fasta (https://www.thegpm.org/crap/) and the sequences of CyaA, pepsin, and nepenthesin-2. Search parameters were set as follows: precursor tolerance 10 ppm, fragment ion tolerance 0.05 Da, decoy search enabled, FDR <1%, IonScore >20, and peptide length >5. Variable modifications - Stearoyl, Myristoleyl, Myristoyl-OH, Myristoyl, Steroyl 18:1, Palmitoleyl, Palmitoyl - were set at Lys.

Trypsin digestion and LC-MS/MS analysis of BSA and HEK293 cell lysate

Bovine serum albumin (Merck Life Sciences) was dissolved in 50 mM HEPES (pH 7.5) and 150 mM NaCl buffer. HEK293 cells were resuspended in a lysis buffer consisting of 100mMtriethylammonium bicarbonate, pH 8.5, and 2% sodium deoxycholate, and denatured in a thermomixer (2000 rpm) for 5 min at 95 °C prior to sonication. Acetone precipitation (70% final concentration, 2 h at -20°C) was used to remove sodium deoxycholate detergent and other salts. The samples were centrifuged (16,100 ×g, 15 min) and protein concentration in the pellet was determined using the Pierce BCA protein assay kit (Thermo Fisher Scientific). Samples (50 pmol/reaction) were diluted with 50 mM ethylmorpholine buffer (pH 8.5) and digested with trypsin (1:50 protease:protein ratio (w/w)) for 12 hours at 37 °C. Prior to the analysis, samples were reduced (10 mM TCEP, 10 min at 65 °C) and alkylated (20 mM IAA, 1 h at RT). The peptides were injected onto a PepMap Neo (5 µm C18, 50 × 0.30 mm, Thermo Scientific) trap cartridge, desalted with 0.1% FA in water and separated on a PepSep (1.5 µmC18, 150 × 0.15 mm) analytical column for 15 min by a water/acetonitrile

gradient (4%−35% (v/v); solvent A: 0.1% FA in water, solvent B: 0.1% FA, 20% water in acetonitrile). The column was kept at 50 °C. Solvents were delivered by the UHPLC system (VanquishTM Neo, Thermo Scientific) at a 1.5 µL/min flow rate. The LC system was directly connected to an ESI source of the timsTOF SCP mass spectrometer (Bruker Daltonics), operating in a data-dependent mode employing PASEF. BSA samples were searched using MASCOT (v 2.7, Matrix Science), using the database with BSA and proteases sequences and common cRAP.fasta. Three search modes were used: tryptic (restriction for Pro and one missed cleavage allowed), semi-tryptic, and no-enzyme. HEK293 cell lysate samples were searched using the PEAKS Studio software v12 against a human UniProt database in no enzyme mode. Other parameters set were: 10 ppm mass tolerance for precursor, and 0.05 Da for fragment ions; the modifications set were variable HexNAc at Asn and fixed Cys carbamidomethylation for alkylated samples.

On-line digestion of myoglobin and haptoglobin 2-2 and LC-MS/MS analysis

Horse myoglobin (Merck Life Sciences) (5 µM) was diluted five times with 50 mM HEPES (pH 7.4), 150 mM NaCl buffer, and mixed with 1 M glycine-HCl (pH 2.3) quench buffer at a 1:1 (v/v) ratio. Human haptoglobin 2-2 (Merck Life Sciences) (10 µM) was diluted (5x) with the same buffer and mixed with 0.5 M glycine (pH 2.3), 4 M urea and 0.25 M TCEP quench buffer at 1:1 (v/v) ratio. The experiments were conducted using the same LC set-up as described for analyses of CyaA toxin. Co-immobilized pepsin-nepenthesin-2 proteolytic column at 200 µL/min flow rate was used for online digestion. To analyze Hp 2-2 samples, PNGase Rc column was connected in series with the proteolytic column for online deglycosylation. The LC-MS/MS data were searched using MASCOT (v 2.7, Matrix Science) and custom-built database with four-times concatenated sequence of horse myoglobin, Hp 2-2, proteases used, PNGase Rc, and common cRAP.fasta. Search parameters were set as follows: precursor tolerance 10 ppm, fragment ion tolerance 0.05 Da, decoy search enabled, FDR <1%, IonScore >20, and peptide length >5. Variable modifications – Asn deamidation was set to account for the PNGase driven deglycosylation of Hp 2-2.

References

1. Osickova, A. *et al.* Acyltransferase-mediated selection of the length of the fatty acyl chain and of the acylation site governs activation of bacterial RTX toxins. *J. Biol. Chem.* **295**, 9268–9280 (2020).
2. Masin, J. *et al.* Negatively charged residues of the segment linking the enzyme and cytolysin moieties restrict the membrane-permeabilizing capacity of adenylate cyclase toxin. *Sci. Rep.* **6**, 29137 (2016).

Supporting Figures

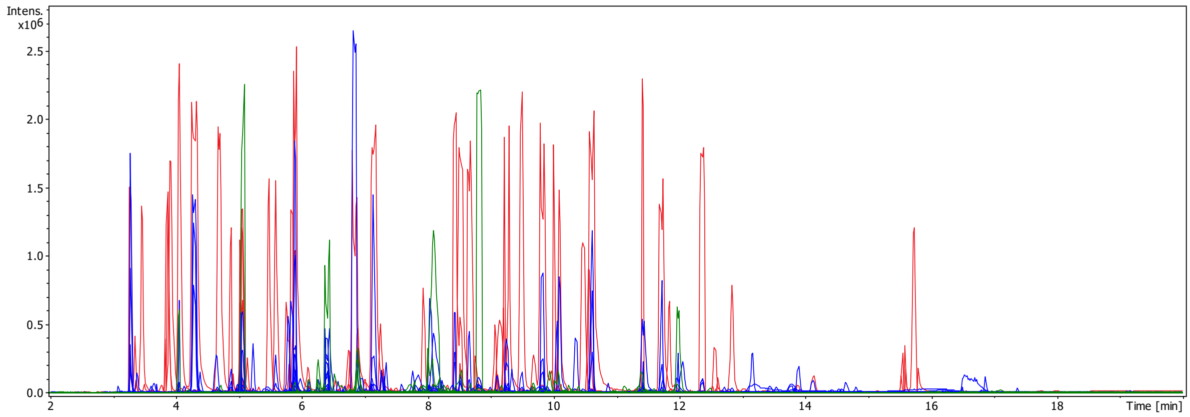

Figure S1. Extracted ion chromatograms of tryptic peptides identified from a bovine serum albumin digest. Reduced and alkylated BSA was digested with trypsin for 12 hours at 37 °C. The data were searched with no enzyme specificity. Extracted ion chromatograms were plotted for all identified peptides. Peptides matching trypsin specificity with up to one missed cleavage are shown in red, those with more than one missed cleavage in green, and semi-specific peptides in blue. No fully non-specific peptides were detected.

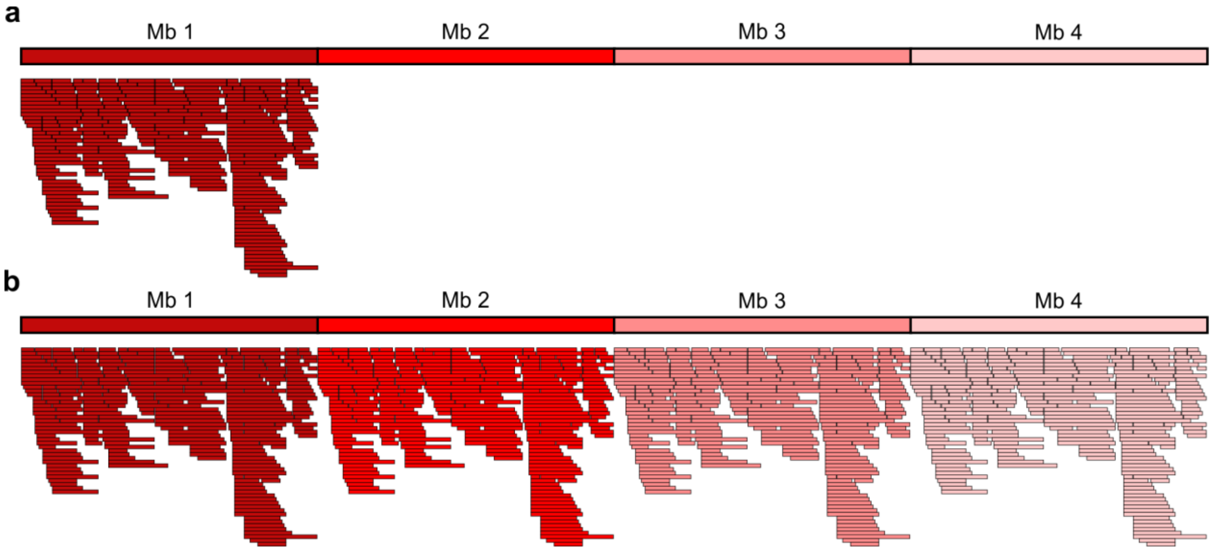

Figure S2. Sequence coverage of concatenated myoglobin. Horse heart myoglobin was digested online using a co-immobilized pepsin and nepenthesin-2 column (70 µL bed volume, 200 µL/min flow rate, 0 °C, 0.4% formic acid in water). The resulting data were searched against a concatenated myoglobin sequence containing four repeats. (a) Sequence coverage as reported by MASCOT. (b) Sequence coverage rendered by DigDig, which identifies and visualizes repetitive peptides. Repetitive peptides are highlighted using a red color gradient.

	MASCOT	PEAKS	Byos	ProLuCID	FragPipe	MaxQuant	novor.cloud	DigDig
repetitions recognised	✗	✗	✗	✗	✗	✗	✗	✓
repetitions covered	✗	✗	✗	✓	✓	✓	✓	✓
visualization	✗	✗	✗	✗	✗	✗	✓	✓
output data	✗	✗	✗	✗	✗	✗	✗	✓

Figure S3. Handling of repetitive peptide sequences by various search engines. Data from a non-specific digestion of myoglobin (see Figure S2 caption for details) were searched against a concatenated myoglobin sequence containing four repeats (4×Mb) using MASCOT, PEAKS Studio, Byos, ProLuCID, FragPipe, MaxQuant, and novor.cloud. The figure summarizes how each search engine or software platform processes and reports repetitive peptide identifications. *Repetitions recognized* means, that the software explicitly searches for the occurrence of a repetitive peptide sequence within the target protein (applicable only to DigDig). *Repetition covered* stands for a situation where the coverage was calculated as complete (thus all repetitions were considered), and *visualization* means whether the software plotted a coverage map showing full sequence coverage and not only ¼ coverage. The *output data* indicates that the software provides export of repetitive sequences into a file for further analysis. Screenshots from the individual search engines are included in the following supplementary figures (Figure S4-8). The only exception is MaxQuant, which does not provide this visualization.

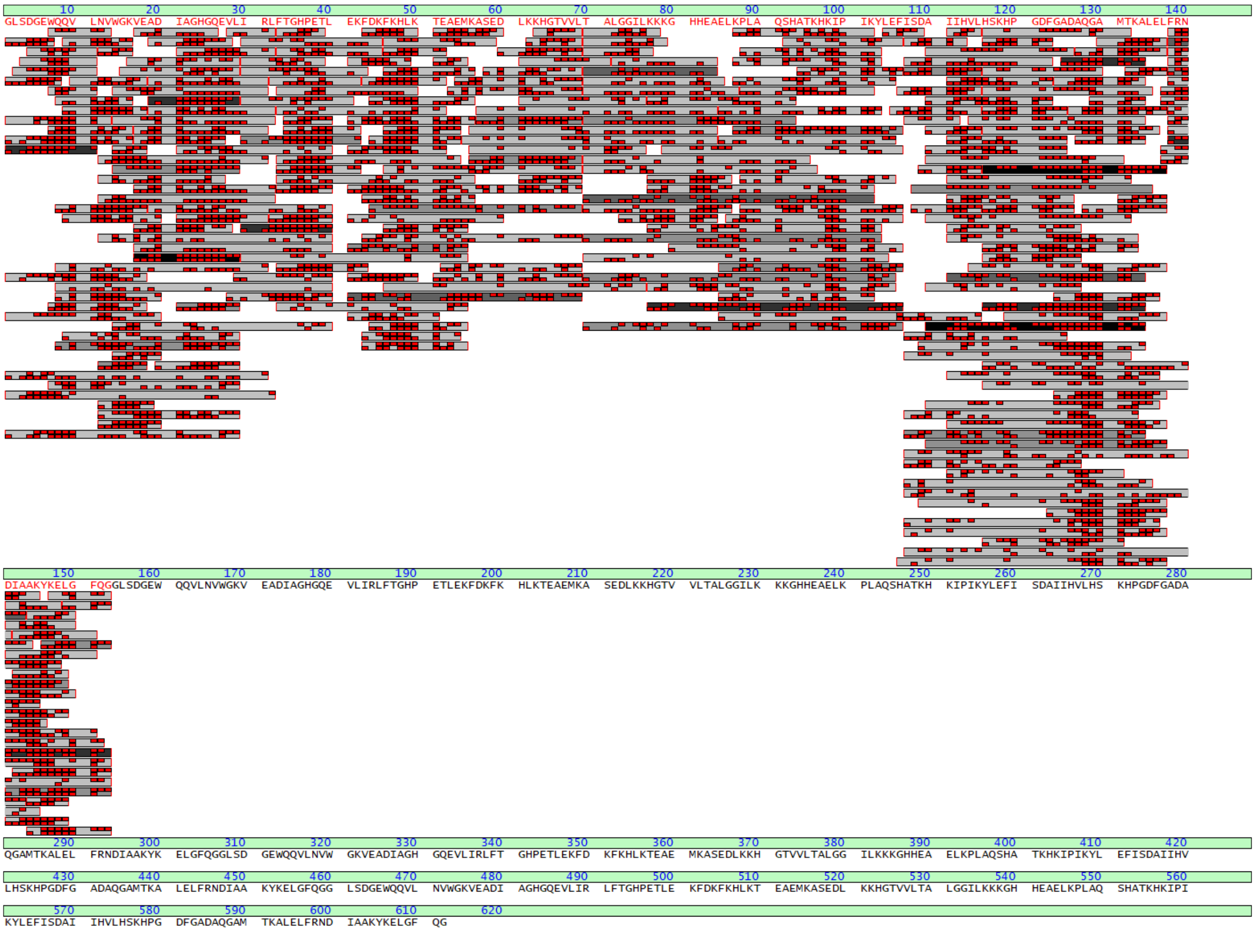

Figure S4. Search result – sequence coverage from MASCOT – BioPharma Compass.

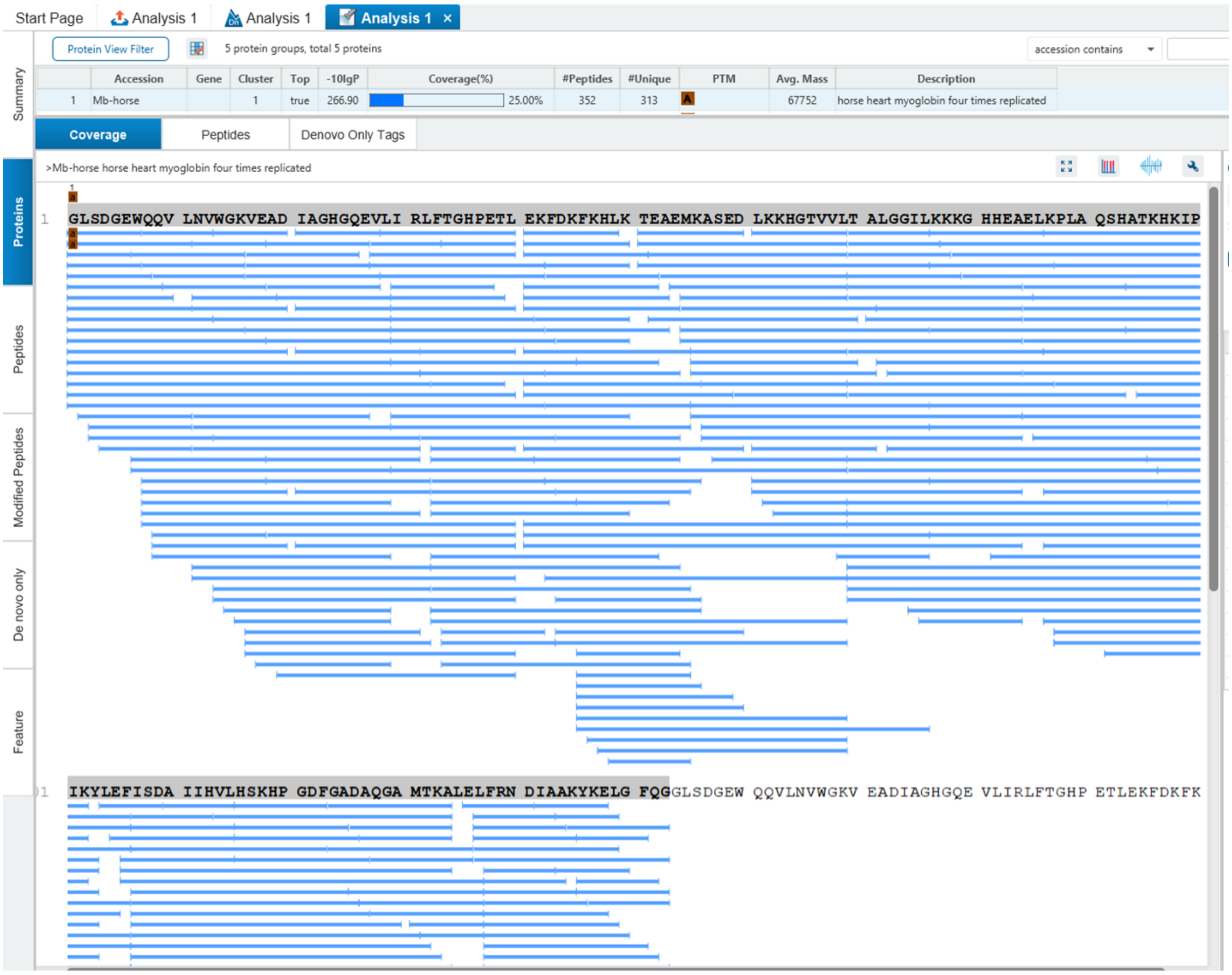

Figure S5. Search result – sequence coverage from PEAKS Studio.

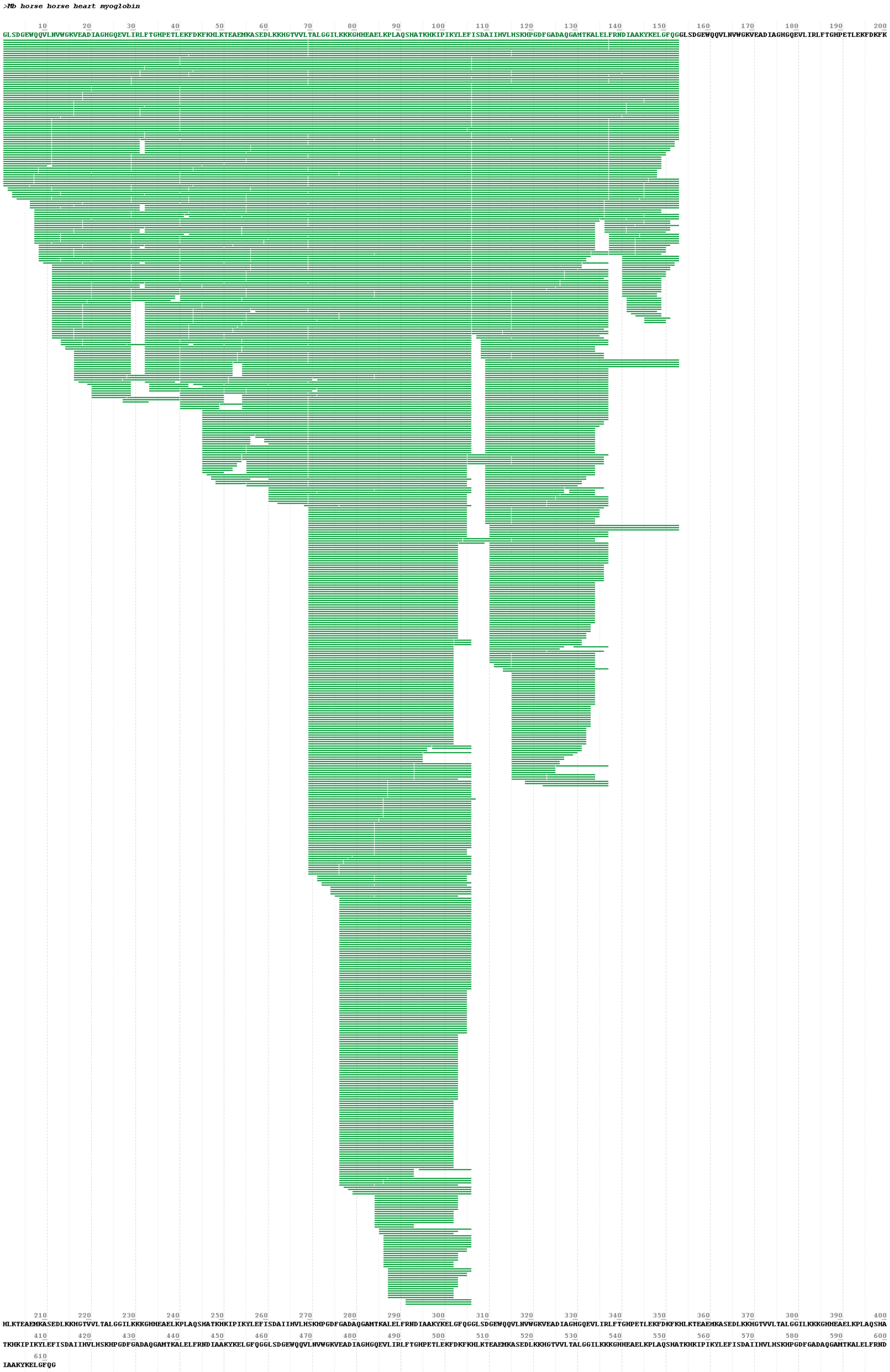

Figure S6. Search result – sequence coverage from Byos.

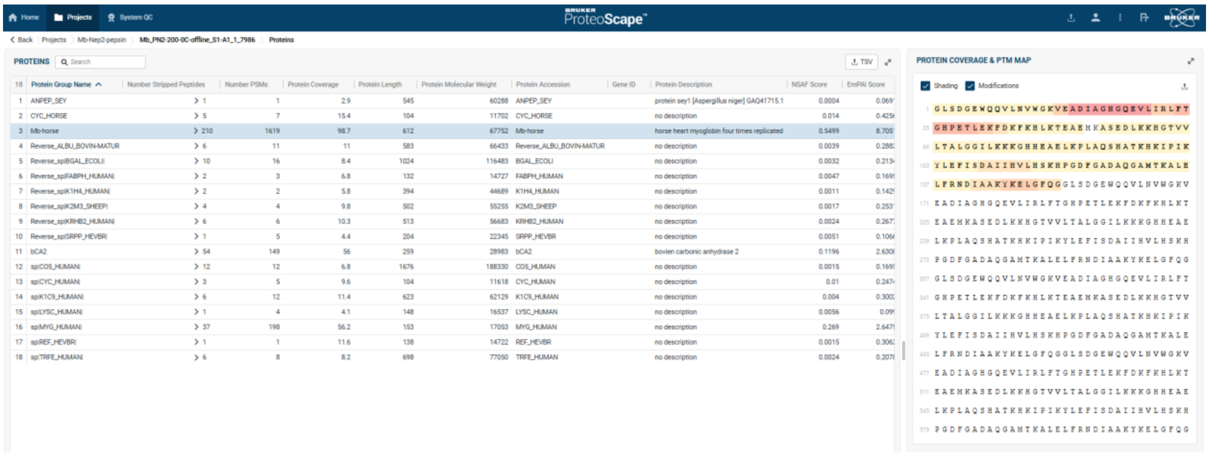

Figure S7. Search result – sequence coverage from ProLuCID - ProteoScape.

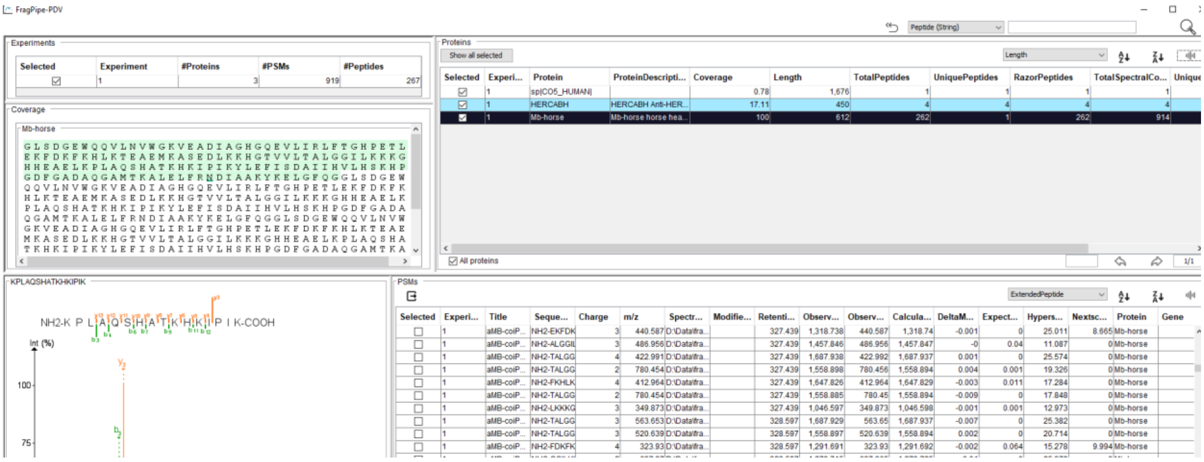

Figure S8. Search result – sequence coverage from FragPipe.

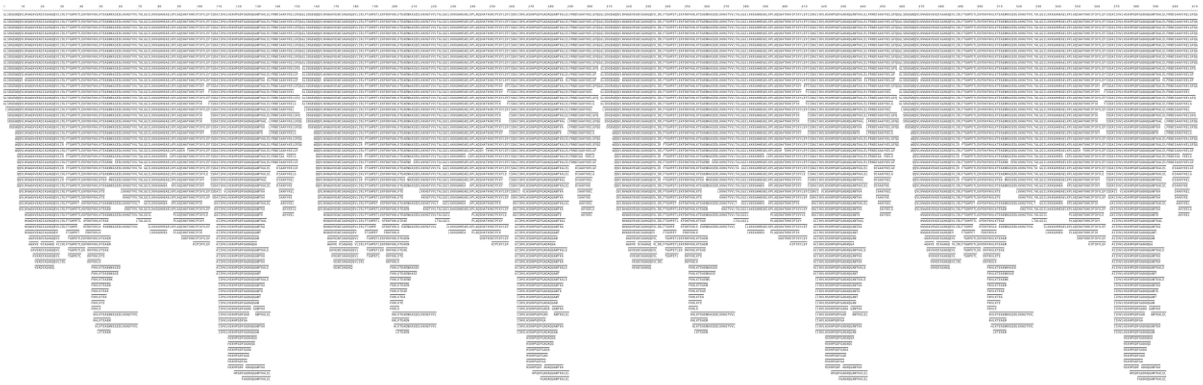

Figure S9. Search result – sequence coverage from novor.cloud. The obvious four repetitions of the coverage profile indicate full coverage of the concatenated myoglobin.
